# Associations between physical activity and autonomic function during deep breathing test: the Swedish CArdioPulmonary bioImage Study (SCAPIS)

**DOI:** 10.1007/s10286-023-00960-y

**Published:** 2023-06-21

**Authors:** Ensieh Memarian, Isabella Kharraziha, Viktor Hamrefors, Pyotr G. Platonov, Örjan Ekblom, Anders Gottsäter, Gunnar Engström

**Affiliations:** 1 Department of Clinical Sciences, Malmö, Internal Medicine Research Group, Skåne University Hospital, Lund University, Jan Waldenströms gata 15, 5th Floor, S-20502 Malmo, Sweden; 2 Department of Cardiology, Skåne University Hospital, Malmö, Sweden; 3 Department of Cardiology, Clinical Sciences Lund, Lund University, Lund, Sweden; 4 Department of Physical Activity and Health, The Swedish School of Sport and Health Sciences, Stockholm, Sweden

**Keywords:** Physical activity, Autonomic function, Deep breathing test, Accelerometer, Heart rate variation, Cardiovagal function

## Abstract

**Purpose:**

The deep breathing test (DBT) is a sensitive test of cardiovagal function. The aim of this study was to explore associations between physical activity and sedentary time, measured by accelerometer, and autonomic function, using DBT.

**Methods:**

In the Swedish Cardio-Pulmonary bioImage Study, men and women aged 50–64 were randomly invited from the general population. A total of 4325 subjects who underwent DBT and assessment of physical activity and sedentary time by accelerometery were included. ECG files from 1-min DBT were used to calculate measures of respiratory sinus arrhythmia [RSA; expiration–inspiration (E-I) difference and E/I ratio], heart rate variability [HRV; root mean square of successive differences (RMSSD), standard deviation of heart rates and mean circular resultant]. Low RSA and HRV was defined as the lowest 10% in the population.

**Results:**

For accelerometer-assessed physical activity, there were significant associations between high percentage of sedentary time and low E/I (*p* < 0.01), and low RMSSD (*p* < 0.01) in an age- and sex-adjusted model, and between percentage of sedentary time and low RMSSD (*p* = 0.04) in a risk factor-adjusted model. Low RMSSD was less common in those with a high percentage of moderate to vigorous physical activity (*p* = 0.04, after risk-factor adjustment). These associations became non-significant when further adjusting for heart rate.

**Conclusion:**

We report associations between degree of physical activity and indices of autonomic dysfunction in a large population. The relationships were no longer significant after adjustments for heart rate, indicating that the relationship between physical activity and cardiovagal function partly is accounted for by reduced heart rate.

**Supplementary Information:**

The online version contains supplementary material available at 10.1007/s10286-023-00960-y.

## Introduction

Dysfunction of the cardiovascular autonomic nervous system has been implicated as a potentially modifiable risk factor for cardiovascular disease (CVD) [1]. The cardiovascular autonomic system can be divided into two major parts, the sympathetic and parasympathetic systems. Changes in heart rate during normal breathing, i.e. respiratory sinus arrhythmia (RSA), are controlled mostly by the parasympathetic nervous system. The deep breathing test (DBT) increases the parasympathetic drive and the heart rate response during RSA [2, 3]. The DBT is therefore a sensitive test of cardiovagal function [2–4]. Low RSA during a DBT is associated with increased occurrence of coronary atherosclerosis in individuals from the general population [4] and with higher mortality in individuals with high cardiovascular risk [5].

A healthy lifestyle, i.e. regular physical activity, avoidance of smoking, moderate consumption of alcohol, and normal body mass index (BMI), has been associated with reduced CVD morbidity and mortality [6, 7]. The pathophysiologic pathways involved are complex, however, and not completely understood. Regular physical activity has positive effects on vagal activity of the heart and thus reduces the negative effects of aging upon the autonomic influence on heart rate [8]. A study by Galetta et al. comparing 10 sedentary healthy elderly and 10 long-distance runners of the same age reported higher parasympathetic function, assessed by DBT and Valsalva test, in the trained subjects compared to the sedentary controls [9]. A recent review reported a positive effect of endurance-type exercise on autonomic function in older adults [10]. These findings may partly be explained by higher aerobic capacity, which includes lower resting heart rate (HR) as a result of larger stroke volume. This lowered HR is primarily a result of increased parasympathetic function [11]. In contrast, a review and meta-analysis of the association between sedentary time and heart rate variability (HRV) found that available data could not support any association between sedentary behaviour and HRV [12]. This is reasonable, since time spent sedentary seems more weakly related to aerobic capacity compared to moderate and vigorous activity [13, 14].

Previous studies have usually studied smaller groups of patients or healthy volunteers, with limited statistical power. Except for the study by Galetta et al. [9], no previous study has used DBT to assess cardiovagal function. Furthermore, many previous studies have used self-reported physical activity rather than accelerometer-based measures, which may possibly explain the inconclusive findings. The aim of this population-based study was to explore associations between physical activity and sedentary time, measured by accelerometer, and autonomic function assessed by a 1-min DBT in a large sample of middle-aged men and women from the general population.

## Materials and methods

### Study population

The Swedish CArdio Pulmonary bioImage Study (SCAPIS) is a nationwide population-based cohort for the study of CVD and chronic obstructive pulmonary disease (COPD). SCAPIS is made possible through a collaborative effort between six Swedish universities and university hospitals [15]. Individuals from the general population, 50–64 years old, were randomly selected and invited by a letter. The exclusion criteria were either not being able to understand instructions in Swedish language or being unable to complete the questionnaires, which was determined by the study staff. The examination took place at the screening centres on three different days, 1–2 weeks apart, during 2014–2018. A total of 30,154 men and women were included in the study. The overall participation rate was 50%.

**Fig. 1:**
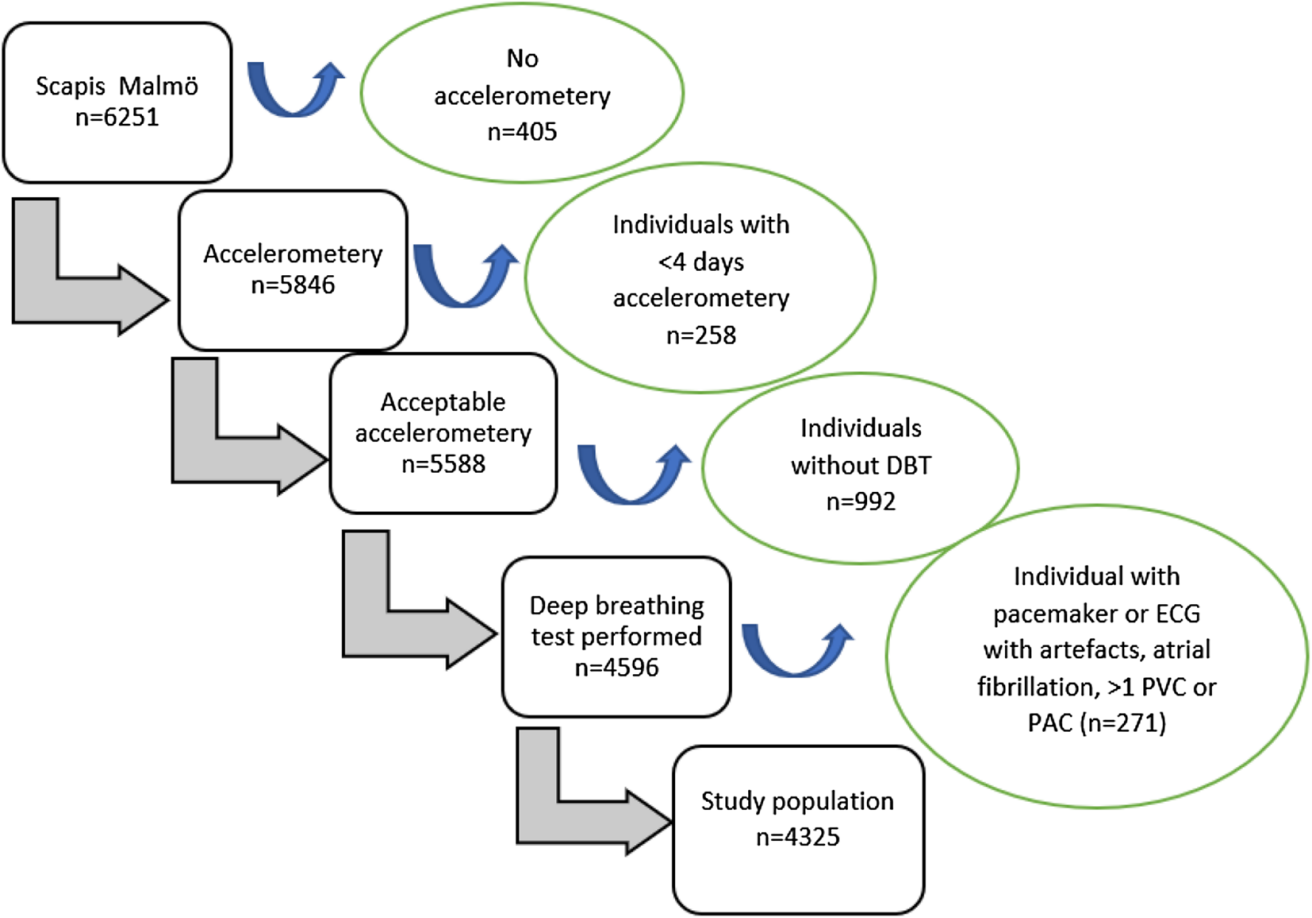
Study population and excluded individuals

The DBT was part of the study protocol for individuals examined at the screening centre in Malmö. An acceptable accelerometry with at least 4 days of registration was performed for 5588 participants, and a 12-lead ECG registration with a DBT was conducted in 5136 out of 6251 participants in Malmö. Information about both DBT and accelerometry was available for 4596 individuals (Fig. 1). The reason for not conducting DBT on all the participants was lack of either time or staff at the time of screening. Mean age (57.5 years versus 57.5 years), gender (men 46.6% versus 48.2%), percentage sedentary time (53% versus 52%) and moderate to vigorous time (6.3% versus 6.4%) were similar between those who did or did not perform DBT. During the first screening day, participants filled in a detailed questionnaire about lifestyle and living conditions and received an accelerometer to wear for 7 days to monitor daily physical activity and sedentary time.

Participants with atrial fibrillation (AF), premature ventricular contractions (PVCs), premature atrial contractions (PACs) or other kinds of ECG artefacts were excluded. We also excluded 12 participants with pacemaker. The final study population consisted of 4325 individuals. A flow chart of study participants and excluded individuals is presented in Fig. 1.

### Basic examination

Information about participants’ lifestyle, including smoking habits, physical activity, sedentary behaviour, and medication for hypertension, was derived from the questionnaire. Smoking was categorized as current smoker (yes or no). Diagnoses for diabetes were based on either subjects’ response in the questionnaire or capillary plasma glucose level of 7 mmol/L or higher.

Body weight was estimated using a digital scale, while the participants were dressed lightly with no shoes on. Height was measured in the standing position with a fixed stadiometer, to the nearest centimetre. Body mass index (BMI) was calculated as weight (kg) divided by the square of the height (m^2^). Both systolic and diastolic blood pressures (SBP and DBP) were measured twice in both arms in the supine position and the arm supported at heart level, with an Omron M10-IT blood pressure reader (Omron Corp, Kyoto, Japan). Blood pressures from the arm with highest mean SBP were used in the analysis.

Blood lipids were analysed using a fasting venous blood sample, with standard methods at the laboratory of Skåne University Hospital.

Since accelerometery was performed during 1 week only, we also used complementary questionnaire data of physical activity and sedentary behaviour during the past year. Physical activity level during leisure time was estimated by the Saltin and Grimby questionnaire of leisure time physical activity [16]. The scale included a question (“How much do you move around and exert yourself physically during your leisure time during past 12 months?”) to which respondents could answer by choosing one of the four response alternatives: 1. Mostly sedentary (e.g. reading, watching TV, etc.), 2. Some light physical activity (e.g. walking or cycling to workplace, more than 4 h /week), 3. Moderate and regular training (e.g. running, swimming etc. at least 2–3 h/week) and 4. Regular hard physical training (e.g. hard training or competition in running, swimming, skiing, etc. at least three times per week).

### Physical activity assessed by accelerometer

Sedentary time and physical activity were derived from sensor-based triaxial accelerometers, ActiGraph model wGT3X-BT (ActiGraph LCC, Pensacola, FL, USA). Participants were instructed to wear the accelerometers for 7 days in a belt around the right hip during waking hours. The only exception was during water-based physical activity. The software ActiLife v.6.13.3 was used to start the accelerometers and for transferring and processing collected data. The movements from three axes were recorded by accelerometer. Accelerometer data was extracted as 60-s epochs and expressed in counts per minute (cpm). Non-wear time was considered when the participants had no movements for 60 or more consecutive minutes. Wear time was estimated as 24 h minus non-wear time. Participants with at least 4 days wear time were included.

Total physical activity was expressed in daily mean cpm. Sedentary time was expressed when activity was less than 200 cpm, low intensity physical activity as 200–2689 cpm, moderate intensity physical activity as 2690–6166 cpm, and vigorous physical activity as 6167 cpm or more. A sedentary time of 20 min or more of below 200 cpm was defined as prolonged sedentary. Detailed information about assessment of physical activity is described by Ekblom-Bak et al. [17].

### ECG and deep breathing test (DBT)

The ECGs were taken in the morning in a tempered and quiet private room with subdued lighting. The subjects were asked to abstain from smoking before visiting the screening centre, but no fasting or other preparations were required. Prior to start of DBT, all procedures were explained to the study subjects. Thereafter, the participants rested for 5 min in the supine position and breathed normally. Then a trained nurse instructed the participants to inhale for 5 s and exhale for 5 s. The nurse used a clock to guide the participants during the inhalation and exhalation manoeuvres. This procedure was repeated during six breathing cycles. Simultaneously, ECGs were registered with a rate of 500 Hz. All the ECGs were screened by two researchers to make sure that no artefacts or ectopic beats distorted the assessment of RSA. ECGs with atrial fibrillation or artefacts, as well as ECGs with more than one premature ventricular or atrial contraction (PVC or PAC) were excluded. One premature contraction during a 1-min recording was accepted and the median-based expiration–inhalation (E-I) difference was utilized for the primary analysis. This measure is robust to single premature contraction [18].

ECG files were used to calculate three measures of RSA, i.e. the median-based expiration–inhalation difference (E-I_median_), mean-based expiration–inhalation difference (E-I_mean_) and E/I ratio (E/I). In the same time domain, measures of HRV were calculated from the same ECGs. The following variables were calculated: standard deviation of heart rate (SD_HR_), root mean square of successive differences (RMSSD) and mean circular resultant (MCR) [18]. MCR is a vector-based measure used to reduce the impact of PVCs and differences in mean heart rate when assessing HRV [19]. The lowest 10% of RSA or HRV in the population was defined as low RSA or HRV. Heart rate and all measures of HRV and RSA were calculated from electronic ECG files according to Löllgen et al. [18], using custom-made software running on MATLAB R2013b (The MathWorks, Inc., Natick, MA, USA) for Linux.

### Test–retest reliability of DBT

A re-examination of 84 individuals from the present cohort was performed after 1 year (± 1 month) and a repeated DBT was performed [4]. The test–retest Spearman correlations for the various measures of HRV and RSA were in the range *r* = 0.59 to 0.69, and the intra-class correlation coefficients were in the range 0.52 to 0.68 (Supplementary Table 1).

### Statistics

The accelerometer-based percentages of moderate to vigorous physical activity and sedentary time, respectively, were divided into quintiles. RSA (i.e. E-I_median_, E-I_mean_, E/I) and HRV (i.e. SD_HR_, RMSSD, MCR) were examined across the quintiles of physical activity and sedentary time. For physical activity and sedentary behaviour assessed by questionnaire, the four categories defined by the Saltin–Grimby questionnaire were used. Logistic regression was used to assess the association between physical activity, sedentary behaviour and measures of RSA and HRV, with low HRV and RSA (i.e. the lowest 10% of the population) as dependent variable. Model 1 was adjusted for age and sex. Model 2 also included adjustments for season, current smoking, diabetes and BMI. Model 3 added heart rate as adjustment variable. The relationship between physical activity, sedentary behaviour and heart rate was analysed in a multiple linear regression model, with heart rate as dependent variable and adjustments for covariates in model 1 and 2.

To further study the association between physical activity and sedentary behaviour and autonomic function, we compared extremes of physical activity, i.e. those with the highest percentage of sedentary time versus those with the highest percentage of moderate/vigorous activity. The top quintile of percentage of sedentary time and moderate/vigorous activity, respectively, was used to define the extreme groups. Furthermore, we compared four groups, defined by sedentary time and moderate/vigorous activity above and below median (i.e. low moderate/vigorous activity and high sedentary time; low moderate/vigorous activity and low sedentary time; high moderate/vigorous activity and high sedentary time; high moderate/vigorous activity and low sedentary time). Linear regression was used to compare continuous variables and logistic regression was used for low measures of DBT.

In a sensitivity analyses, logistic regressions were performed separately for those with heart rate above and below median. Finally, we examined the associations between quintiles of percentage sedentary time and moderate to vigorous physical activity, respectively, with continuous measures of DBT as dependent variables using three linear regression models.

IBM SPSS Statistics (v. 27, Armonk, NY, USA) software was used for all statistical calculations.

## Results

Characteristics of the study population in relation to the accelerometer-based percentage of sedentary time are presented in Table 1. The most sedentary individuals (Q5) were more often men, smokers, had higher blood pressure, BMI, heart rate, and higher prevalence of type 2 diabetes compared to those least sedentary (Q1). Associations between low measures of DBT and percentage sedentary time were analysed in three logistic regression models. There were significant associations between high percentage sedentary time and low E-I ratio (*p* < 0.01), low SD (*p* = 0.03), and low RMSSD (*p* < 0.01) after adjustment for age and sex in model 1, and between sedentary time and low RMSSD (*p* = 0.04) after adjustment for risk factors in model 2. These relationships were non-significant after further adjustment for heart rate in model 3. There was a significant relationship between high percentage of sedentary time and high heart rate. This relationship remained significant after adjustment for covariates in model 1 and 2 (Table 1).

**Table 1:** Associations between percentage of sedentary time measured by accelerometer, study characteristics, and measures of the DBT

	% sedentary time					P1^a^	P2^b^	P3^c^
	Lowest				Highest			
Total *N* = 4325	Q1 (*n* = 820)	Q2 (*n* = 982)	Q3 (*n* = 739)	Q4 (*n* = 909)	Q5 (*n* = 875)			
% sedentary time	37.6	47.2	53.0	58.4	66.9			
Age (years)	57.3 ± 4.3	57.7 ± 4.3	57.4 ± 4.3	57.3 ± 4.3	57.6 ± 4.2			
Women (%)	67	61	53	51	43			
Systolic blood pressure (mmHg)	123 ± 17	122 ± 16	122 ± 16	124 ± 17	125 ± 17			
Diastolic blood pressure (mmHg)	74.8 ± 10	74.5 ± 9.1	74.8 ± 9.6	75.9 ± 9.8	76.7 ± 9.9			
Smoking (%)	15.6	16.4	15.0	15.3	17.6			
Diabetes (%)	5.9	6.1	7.9	7.8	11.9			
BMI (kg/m^2^)	25.9 ± 4.0	26.5 ± 4.1	26.8 ± 4.0	27.5 ± 4.5	28.8 ± 5.2			
LDL (mmol/l)	3.5 ± 0.94	3.6 ± 0.91	3.6 ± 0.94	3.6 ± 0.97	3.6 ± 0.99			
Heart rate (bpm)	63.4 ± 8.1	63.6 ± 8.9	62.7 ± 8.7	63.7 ± 9.0	64.3 ± 9.4	< 0.01	0.018	
Low E-I median (%)	9.6	9.8	9.2	10.8	12.0	0.17	0.51	0.37
Low E-I mean (%)	9.3	8.9	9.5	10.0	12.1	0.13	0.25	0.14
Low E-I ratio (%)	7.6	7.3	7.0	8.8	11.1	< 0.01	0.16	0.24
Low SD (%)	9.4	9.8	9.9	10.9	13.1	0.03	0.15	0.08
Low MCR (%)	9.8	12.7	8.4	8.9	9.0	0.09	0.08	0.09
Low RMSSD (%)	7.6	9.1	8.7	9.4	12.6	< 0.01	0.04	0.37

Logistic regression was used to analyse low E-I, low SD etc., defined as lowest 10% of the distribution, with adjustment for covariates

^a^P1* p* value adjusted for age and sex

^b^P2 adjusted for age, sex, season, current smoking, diabetes, and BMI

^c^P3 adjusted for age, sex, season, current smoking, diabetes, BMI, and heart rate

For continuous measures of DBT there were no significant associations with percentage of sedentary time (Supplementary Table 2).

Individuals with a high percentage of moderate to vigorous physical activity were younger, more often men, non-smokers, had lower blood pressure, lower BMI and heart rate, as well as lower prevalence of diabetes type 2 compared to those with a low percentage of moderate and vigorous activity (Table 2). The relationship between moderate to vigorous activity and proportion with low DBT measures is presented in Table 2 and results for continuous measures of the DBT are given in Supplementary Table 3. Moderate to vigorous activity was significantly associated with low RMSSD after adjustment for risk factors (*p* = 0.04) but no associations for measures of the DBT remained significant after further adjustment for heart rate. However, low measures of DBT showed a U-shaped association with percentage of moderate to vigorous activity; the proportions with low DBT indices were lower in Q2–Q4, compared to Q1 or Q5. Individuals with a high percentage of moderate to vigorous activity had lower heart rate. This was significant (*p* < 0.001) even after adjustment for covariates in model 2 (Table 2).

**Table 2:** Percentage of time spent in moderate to vigorous activity assessed by accelerometer, characteristics of the study population, and measures of the DBT

	% moderate to vigorous activity time					P1^a^	P2^b^	P3^c^
	Least				Most			
Total *N* = 4325	Q1 (*n* = 872)	Q2 (*n* = 587)	Q3 (*n* = 1101)	Q4 (*n* = 844)	Q5 (*n* = 921)			
% moderate or vigorous time	2.4	4.0	5.5	7.4	11.3			
Age (years)	58.3 ± 4.2	57.4 ± 4.3	57.4 ± 4.3	57.2 ± 4.2	57.1 ± 4.3			
Women (%)	58	61	58	52	46			
Systolic blood pressure (mmHg)	126 ± 17	123 ± 17	123 ± 17	121 ± 16	122 ± 16			
Diastolic blood pressure (mmHg)	76.6 ± 9.6	76.0 ± 10	75.3 ± 9.9	74.4 ± 9.3	74.7 ± 9.4			
Smoking (%)	24.9	17.2	15.1	13.4	10.4			
Diabetes (%)	12.4	6.5	7.4	6.3	6.5			
BMI (kg/m^2^)	28.1 ± 5.0	27.3 ± 4.5	26.9 ± 4.4	26.7 ± 4.2	26.7 ± 4.3			
LDL (mmol/l)	3.6 ± 0.99	3.7 ± 0.95	3.6 ± 0.96	3.6 ± 0.93	3.6 ± 0.92			
Heart rate (bpm)	65.6 ± 9.6	63.8 ± 8.2	63.4 ± 8.9	63.1 ± 8.7	62.2 ± 8.4	< 0.01	< 0.01	
Low E-I median (%)	12.3	8.2	10.1	8.4	11.8	0.99	0.61	0.96
Low E-I mean (%)	11.6	7.8	9.9	9.1	10.5	0.42	0.70	0.26
Low E-I ratio (%)	11.5	7.2	7.6	7.2	8.3	0.03	0.17	0.36
Low SD (%)	12.7	9.4	10.3	9.0	11.4	0.30	0.66	0.26
Low MCR (%)	11.9	7.7	8.7	8.9	11.6	0.51	0.60	0.76
Low RMSSD (%)	14.8	7.0	7.7	9.2	8.4	< 0.01	0.04	0.90

Logistic regression was used to analyse low E-I, low SD etc., defined as lowest 10% of the distribution, with adjustment for covariates. Linear regression was used to analyse heart rate, with adjustments for covariates

^a^P1* p* value adjusted for age and sex

^b^P2 adjusted for age, sex, season, current smoking, diabetes, and BMI

^c^P3 adjusted for age, sex, season, current smoking, diabetes, BMI, and heart rate

For continuous measures of DBT variables, the associations for E-I ratio (*p* = 0.018) and RMSSD (*p* < 0.001) were significant in model 2 but not after adjustment for heart rate (Supplementary Table 3).

Table 3 presents the results for physical activity and sedentary behaviour based on questionnaire. The most active individuals were younger, more often men, had lower blood pressure, BMI, LDL, were more often non-smokers, and had lower prevalence of diabetes type 2. For measures of low DBT, associations were significant for low E-I ratio (*p* = 0.048) and low RMSSD (*p* < 0.001) in model 2, but no longer significant after adjustment for heart rate. Individuals who reported regular training in the questionnaire had significantly lower heart rate than those who were mostly sedentary (Table 3).

**Table 3:** Associations between physical activity and sedentary behaviour according to questionnaire, study population characteristics, and measures of the DBT in regression models

	Least			Most	P1^a^	P2^b^	P3^c^
	0	1	2	3			
	Mostly sedentary	Some light physical activity	Moderate regular training	Regular hard physical training			
Number (*N* = 4123)	570	2034	1079	440			
Age (years)	57.3 ± 4.3	57.9 ± 4.2	57.4 ± 4.3	56.0 ± 4.1			
Women (%)	49	59	55	45			
% sedentary time (accelerometer)	56 ± 11	52 ± 10	52 ± 10	51 ± 10			
% moderate to vigorous time (accelerometer)	4.6 ± 2.9	6.1 ± 3.2	7.0 ± 3.2	7.8 ± 3.8			
Systolic blood pressure (mmHg)	126 ± 17	124 ± 16	122 ± 16	119 ± 16			
Diastolic blood pressure (mmHg)	77.0 ± 9.6	75.9 ± 9.6	74.5 ± 9.5	72.2 ± 9.8			
Smoking (%)	25	18	11	8.4			
Diabetes (%)	12	9.1	3.6	3.6			
BMI (kg/m^2^)	28.9 ± 5.0	27.4 ± 4.6	25.9 ± 3.9	25.6 ± 3.7			
LDL (mmol/l)	3.6 ± 1.0	3.6 ± 0.97	3.6 ± 0.91	3.5 ± 0.88			
Heart rate (bpm)	65.1 ± 9.5	64.6 ± 8.8	62.2 ± 8.4	60.4 ± 7.9	< 0.01	< 0.01	
Low E-I median (%)	11.8	10.1	10.0	8.9	0.47	0.86	0.57
Low E-I mean (%)	12.1	9.9	8.8	9.1	0.18	0.48	0.09
Low E-I ratio (%)	11.4	8.6	7.4	4.8	< 0.01	0.05	0.16
Low SD (%)	11.8	10.9	9.2	9.8	0.26	0.86	0.29
Low MCR (%)	11.8	9.6	8.6	9.1	0.16	0.60	0.44
Low RMSSD (%)	13.7	10.8	6.0	6.6	< 0.01	< 0.01	0.25

Logistic regression was used to analyse low E-I, low SD etc., defined as lowest 10% of the distribution, with adjustment for covariates. Individuals not answering the questionnaire were excluded (*n* = 202)

^a^P1* p* value adjusted for age and sex

^b^P2 adjusted for age, sex, season, current smoking, diabetes, and BMI

^c^P3 adjusted for age, sex, season, current smoking, diabetes, BMI, and heart rate

For continuous measures of DBT variables in relation to physical activity according to questionnaire, the associations for E-I median (*p* = 0.04), E-I mean (*p* = 0.044), and RMSSD (*p* = 0.035) were significant in model 2 but not after adjustment for heart rate (Supplementary Table 4).

In Supplementary Table 5 extremes of accelerometry, i.e. the most sedentary individuals, are compared with those with most moderate and vigorous physical activity. Significant associations between physical activity and DBT measures occurred in model 2 for E-I median (*p* = 0.014) and E-I mean (*p* = 0.022) in linear regression. For measures of low DBT, high physical activity was associated with low MCR in all models (*p* = 0.041 in model 3).

We also compared four groups defined by moderate/vigorous activity and sedentary time above and below median. Again, we found significant differences in heart rate between the physically active groups, but with the exception of MCR, measures of DBT were non-significant after multivariate adjustments (Supplementary Table 6).

Since adjustment for heart rate markedly attenuated the relationship between DBT measures and physical activity time and sedentary time, respectively, analyses were repeated for individuals with heart rate above and below the median (i.e. 63 bpm). In participants with heart rate below median, no significant relations were observed. In participants with heart rate above median, both low RMSSD and low E-I ratio were related to time spent sedentary (Table 4).

**Table 4:** Odds ratios (OR) and* p* values for relationships between % sedentary time in individuals with heart rate above and below median (63 bpm)

	Heart rate below median (*n* = 2192)			Heart rate above median (*n* = 2133)		
	Sedentary time			Sedentary time		
	OR	95% CI	*p* value	OR	95% CI	*p* value
Low E-I median (%)	0.79	0.21–3.0	0.73	3.1	0.66–14	0.15
Low E-I mean (%)	1.71	0.46–6.4	0.42	2.1	0.41–11	0.37
Low E-I ratio (%)	0.38	0.07–2.1	0.27	6.7	1.5–29	0.01
Low SD (%)	1.30	0.35–4.6	0.72	4.3	0.89–21	0.07
Low MCR (%)	0.56	0.14–2.3	0.42	0.29	0.07–1.2	0.09
Low RMSSD (%)	0.67	0.06–7.1	0.74	3.5	1.1–11	0.04

Adjusted for age, sex, season, current smoking, diabetes, and BMI. Dependent variables were low RSA or HRV from deep breathing test (i.e. lowest 10% of the population). ORs are expressed as proportion of sedentary time out of total time wear time

In Supplementary Table 7, odds ratios (ORs) and* p* values with mutual adjustments for % sedentary time and % moderate to vigorous physical activity time are presented.

## Discussion

Previous studies of physical activity and autonomic function [8–14] have shown inconsistent results. This could potentially be explained by methodological differences in assessment of autonomic function as well as type of measurement and the grading of the intensity of physical activity. We examined the relationship between cardiovagal function assessed by DBT and physical activity and sedentary behaviour assessed by accelerometer as well as a validated questionnaire, in a large middle-aged population-based sample. We found a significant association between low RMSSD, a marker of reduced cardiovagal function, and high percentage of sedentary time after adjustment for several relevant confounders such as age, sex, season, current smoking, diabetes, and BMI. This association disappeared, however, when adjusted for heart rate. Similarly, we found significant associations between moderate to vigorous physical activity and E-I_median_, E-I_mean_, and RMSSD when adjusted for age, sex, season, current smoking, diabetes, and BMI. These associations were no longer detectable when adjusting for heart rate. The lack of significant associations in our study is largely in concordance with results from a recent meta-analysis by Alansare et al. reporting that there is no independent association between sedentary time and HRV [12]. On the other hand, a meta-analysis by Raffin et al. showed a positive effect of exercise on autonomic regulation in older adults and the authors concluded that exercise leads to HRV improvements, although they also identified potential bias in the included studies [10]. Another recent study showed that accelerometer-estimated physical activity, both vigorous and light-intensity, was associated with improved HRV [20]. The fact that the associations between markers of autonomic function and physical activity disappeared in our study, after adjusting for heart rate, might be explained by effects of physical activity on heart rate, which can be regarded as a measure of cardiovagal function. Thus, one may argue that the adjustment for heart rate could be considered as overadjustment. On the other hand, our current results indicate that the effects of physical activity on autonomic function could be estimated by the simple measurement of resting heart rate, rather than by more advanced tests such as DBT [21, 22]. As such, our results are in accordance with the hypothesis proposed by Monfredi et al. [23], who suggested that the correlation between HRV and altered morbidity and mortality substantially could be attributed to correlations with heart rate.

Although increased parasympathetic drive is associated with increased HRV, this relationship is not completely linear and could vary between individuals and situations. It has been reported that increased parasympathetic stimulation could lead to a plateau, or a saturation effect, above which HRV will be stable or even decrease [24, 25]. The present study found significant relationships between high percentage of sedentary time and low RMSSD and E/I ratio for those with heart rate above median, but no relationship for those with low heart rate. This could possibly indicate that some participants with low heart rate had reached this plateau.

Accelerometer-based assessments of physical activity usually cover a shorter period; in this study 7 days of registration was used. In contrast, the physical activity questionnaire in this study covered the past 12 months and it is possible that longer periods of physical activity are necessary to induce physiological effects on autonomic function. It is noteworthy that the relationship between heart rate and physical activity and sedentary behaviour assessed by questionnaire was comparatively strong. There was a significant association between having low E/I and low RMSSD and being mostly sedentary in leisure time according to questionnaire. The associations disappeared, however, when adjusted for heart rate in model 3 and the overall results after multivariate adjustments were similar for physical activity and sedentary behaviour based on questionnaire or accelerometer, respectively. A systematic review from 2021 assessing physical activity and HRV reported that higher intensities and frequencies of physical activity might enhance HRV, cardiovascular health, and risk factors [20]. However, quality assessment of studies included in this systematic review unveiled both methodological and reporting deficits.

We found that a sedentary lifestyle was associated with other risk factors such as higher blood pressure, BMI, and diabetes prevalence compared to a more physically active lifestyle. We thereby corroborate previously presented results regarding relationships between low physical activity and higher blood pressure [26], diabetes and adiposity [27, 28]. In our study individuals with the highest self-reported physical activity had lower blood pressure, BMI, LDL, and prevalence of diabetes.

Cardiovagal dysfunction has been associated with increased risk of atherosclerosis and CVD. One important question is whether cardiovagal dysfunction could be improved by lifestyle interventions. A longitudinal cohort study concluded that a number of unhealthy factors such as physical inactivity, smoking, and high BMI were associated with lower HRV and autonomic dysfunction [29] and that a decreasing number of healthy lifestyle practices was associated with subsequent lower measures of cardiovagal function. Our overall findings suggest that the relationships between physical activity, sedentary behaviour and cardiovagal function are weak and, if anything, mediated via effects on heart rate. Whether physical activity could add positive effects on cardiovagal function in addition to other lifestyle interventions should be examined in further studies. It should also be noted that the DBT measures the parasympathetic part on the autonomic nervous system and it is possible that physical activity also has positive effects on cardiovascular health through effects on the sympathetic part of the autonomic system.

## Limitations and strengths

The most important strength of this study is the large population-based sample with information about DBT and physical activity and sedentary behaviour. A previous study from the present cohort showed that results from the DBT are strongly related to coronary atherosclerosis [4]. We also showed that DBT results, in terms of 1-year test–retest reliability, have acceptable stability over time in this middle-aged population (Supplementary Table 1 [4]). The assessment of physical activity and sedentary time by accelerometery over 1 week is also a major strength. The proportion of subjects excluded as a result of less than 4 days of registration was acceptable.

The present study has several limitations. The study population is 50–64 years old, and it is therefore uncertain whether study results are applicable to older or younger age groups. The DBT is commonly performed once during a 1-min period, as in this study, but average values from several consecutive 1-min DBT would possibly have given better estimates of the actual values. There was no previous familiarization of the DBT for the participants and lack of familiarization of this test might be a limitation of this study. However, the procedures were explained prior to the test, the DBT is comparatively easy to perform and the breathing manoeuvres were guided by a nurse. As this study is cross-sectional, we cannot evaluate possible causal relationships. Another limitation of this study is that food and fluid intake were not controlled for before the cardiac-autonomic assessment. Hence, the tests were taken in a non-fasting state, potentially modifying the DBT values.

## Conclusion

A sedentary lifestyle as measured by accelerometer was related to indices of autonomic dysfunction in a large sample of middle-aged individuals from the background population. The associations between degree of physical activity and indices of cardiovagal dysfunction were attenuated and no longer significant after adjustment for heart rate, thereby indicating that reduced heart rate might partly explain the relationships between physical activity and cardiovagal function. Prospective follow-up of the cohort will help clarify the importance of physical activity and autonomic dysfunction for development of cardiovascular disease and events.

## Supplementary Information

Below is the link to the electronic supplementary material.

Supplementary file1 (DOCX 35 KB)
