# Supplementary material for: Associations between physical activity and autonomic function during deep breathing test: the Swedish CArdioPulmonary bioImage Study (SCAPIS)
Source: Clin Auton Res. 2023 Jun 21;33(4):411–20. doi: 10.1007/s10286-023-00960-y (PMC10439237; doi:10.1007/s10286-023-00960-y)
Supplement: Supplementary file 1 — Supplementary file1 (DOCX 35 KB) [file 10286_2023_960_MOESM1_ESM.docx]

Supplementary table 1. One-year test-retest reliability of respiratory sinus arrhythmia and heart rate variability measures for 84 subjects with a repeated deep breathing test after one year (+/- one month).

|  | Baseline  (mean+SD) | Re-exam, 1 year (mean+SD) | P | Spearman r | ICC |
| --- | --- | --- | --- | --- | --- |
| E-I_median_ (bpm) | 10.2±5.5 | 9.8±4.7 | 0.47 | 0.64 | 0.57 |
| E-I_mean_ (bpm) | 10.8±5.4 | 10.5±4.9 | 0.47 | 0.61 | 0.58 |
| E/I | 1.19±0.12 | 1.17±0.10 | 0.17 | 0.69 | 0.53 |
| SD (bpm) | 4.34±2.0 | 4.15±1.8 | 0.35 | 0.59 | 0.54 |
| MCR | 2.24±1.1 | 2.12±1.0 | 0.30 | 0.59 | 0.52 |
| RMSSD (ms) | 54.3±46.7 | 52.0±43.6 | 0.57 | 0.66 | 0.68 |
| Heart rate (bpm) | 62.0±8.7 | 64.1±10.4 | 0.02 | 0.68 | 0.67 |

_P-value refers to difference in mean value between baseline examination and re-examination. ICC Intra-class correlation coefficient_

Supplementary table 2. Associations between percentage sedentary time, measured by accelerometer and continuous measures of the DBT, in three linear regression models

|  | %sedentary time | | | |  |  |  |  |
| --- | --- | --- | --- | --- | --- | --- | --- | --- |
|  | best |  |  |  | worst | P 1^*^ | P 2^**^ | P3^***^ |
| Total N=4325 | Q1 (n=820) | Q2 (n=982) | Q3 (n=739) | Q4 (n=909) | Q5 (n=875) |  |  |  |
| %sedentary | 37.6 | 47.2 | 53.0 | 58.4 | 66.9 |  |  |  |
| E-I median | 10.7±5.4 | 10.7±5.6 | 10.7±5.8 | 10.8±6.0 | 10.7±6.1 | 0.78 | 0.23 | 0.44 |
| E-I mean | 11.5±5.5 | 11.5±5.5 | 11.6±5.8 | 11.6±5.9 | 11.4±6.0 | 0.94 | 0.31 | 0.54 |
| E-I ratio | 1.19±0.11 | 1.19±0.11 | 1.19±0.12 | 1.19±0.12 | 1.19±0.12 | 0.89 | 0.37 | 0.31 |
| SD | 4.5±2.0 | 4.6±2.1 | 4.6±2.1 | 4.6±2.2 | 4.5±2.2 | 0.81 | 0.37 | 0.61 |
| MCR | 1.95±1.20 | 1.93±1.25 | 2.02±1.24 | 2.00±1.29 | 1.94±1.20 | 0.35 | 0.14 | 0.23 |
| RMSSD | 53.3±33 | 55.2±37 | 57.5±41 | 54.2±38 | 54.0±39 | 0.58 | 0.76 | 0.20 |

_Continuous measures of the deep breathing test were used as dependent variable in linear regression models._

_*P 1 p-value adjusted for age and sex_

_**P 2 adjusted for age, sex, season, current smoking, diabetes, and BMI_

_***P 3 adjusted for age, sex, season, current smoking, diabetes, BMI, and heart rate_

|  | % moderate and vigorous activity | | | | |  |  |  |
| --- | --- | --- | --- | --- | --- | --- | --- | --- |
|  | Least |  |  |  | Most | P 1^*^ | P 2^**^ | P3^***^ |
| Total 4325 | Q1 (n=872) | Q2 (n=587) | Q3 (n=1101) | Q4 (n=844) | Q5 (n=921) |  |  |  |
| % moderate or vigorous | 2.4 | 4.0 | 5.5 | 7.4 | 11.3 |  |  |  |
|  |  |  |  |  |  |  |  |  |
| E-I median (bpm) | 10.4±5.8 | 11.4±6.1 | 10.8±5.7 | 10.9±6.0 | 10.3±5.4 | 0.13 | 0.040 | 0.37 |
| E-I mean (bpm) | 11.2±5.8 | 12.1±6.0 | 11.6±5.7 | 11.6±5.8 | 11.1±5.4 | 0.15 | 0.044 | 0.35 |
| E-I ratio | 1.18±0.11 | 1.20±0.12 | 1.19±0.11 | 1.20±0.12 | 1.19±0.11 | 0.86 | 0.70 | 0.48 |
| SD (bpm) | 4.4±2.1 | 4.7±2.2 | 4.6±2.1 | 4.6±2.1 | 4.4±2.0 | 0.26 | 0.064 | 0.39 |
| MCR | 1.88±1.23 | 2.06±1.25 | 2.00±1.22 | 2.01±1.26 | 1.91±1.23 | 0.34 | 0.22 | 0.65 |
| RMSSD (bpm) | 50.5±38 | 55.7±35 | 55.3±37 | 56.0±38 | 56.6±39 | 0.01 | 0.035 | 0.80 |

Supplementary table 3. Association between percentage time spent in moderate and vigorous activity, measured by accelerometer, and continuous measures of the DBT, in three linear regression models

^Continuous measures of the deep breathing test were used as dependent variable in linear regression models.^

^*P 1 p-value adjusted for age and sex^

^**P 2 adjusted for age, sex, season, current smoking, diabetes, and BMI^

^***P 3 adjusted for age, sex, season, current smoking, diabetes, BMI, and heart rate^

Supplementary table 4. Association of physical activity patterns according to questionnaire and continuous measures of DBT, in three linear regression models.

|  | Least |  |  | Most | P 1^*^ | P 2^**^ | P3^***^ |
| --- | --- | --- | --- | --- | --- | --- | --- |
|  | 0 | 1 | 2 | 3 |  |  |  |
|  | Mostly sedentary | Light physical activity | Moderate regular training | Regular hard physical training |  |  |  |
| N 4123 | 570 | 2034 | 1079 | 440 |  |  |  |
| E-I median (bpm) | 10.4±5.9 | 10.7±5.7 | 10.7±5.7 | 11.1±5.4 | 0.44 | 0.98 | 0.13 |
| E-I mean (bpm) | 11.3±5.9 | 11.5±5.7 | 11.5±5.8 | 11.9±5.6 | 0.29 | 0.94 | 0.13 |
| E-I ratio | 1.18±0.12 | 1.19±0.11 | 1.19±0.12 | 1.21±0.12 | <0.001 | 0.018 | 0.064 |
| SD (bpm) | 4.4±2.1 | 4.5±2.2 | 4.5±2.1 | 4.7±2.1 | 0.15 | 0.81 | 0.12 |
| MCR | 1.92±1.24 | 1.96±1.22 | 2.01±1.26 | 2.04±1.23 | 0.33 | 0.82 | 0.43 |
| RMSSD (bpm) | 53.3±42 | 51.5±33 | 58.2±41 | 64.1±42 | <0.001 | <0.001 | 0.21 |

^Continuous measures of the deep breathing test were used as dependent variable in linear regression models.^

^*P 1 p-value adjusted for age and sex^

^**P 2 adjusted for age, sex, season, current smoking, diabetes, and BMI^

^***P 3 adjusted for age, sex, season, current smoking, diabetes, BMI, and heart rate^

Supplementary table 5. Extremes of accelerometry based physical activity and sedentary time by study population characteristics and measures of DBT

|  | Least | Most | P 1^*^ | P 2^**^ | P3^***^ |
| --- | --- | --- | --- | --- | --- |
|  | Most sedentary quintile (Q5 of %sedentary time) | Most active quintile (Q5 of moderate/ vigorous time) |  |  |  |
| Number | 827 | 873 |  |  |  |
| Age (year) | 57.6±4.2 | 57.1±4.3 |  |  |  |
| Women (%) | 43 | 48 |  |  |  |
| Systolic blood pressure (mmHg) | 125±17 | 122±16 |  |  |  |
| Diastolic blood pressure (mmHg) | 76.6±10 | 74.8±9.5 |  |  |  |
| Smoking (%) | 18 | 10 |  |  |  |
| Diabetes (%) | 12.3 | 6.3 |  |  |  |
| BMI (kg/m^2^) | 28.7±5.2 | 26.6±4.2 |  |  |  |
| LDL (mmol/l) | 3.6±0.98 | 3.6±0.92 |  |  |  |
| Heart rate (bpm) | 64.5±9.5 | 62.2±8.4 | <0.001 | <0.001 |  |
|  |  |  |  |  |  |
| E-I median (bpm) | 10.7±6.2 | 10.4±5.5 | 0.10 | 0.014 | 0.11 |
| E-I mean (bpm) | 11.5±6.0 | 11.2±5.5 | 0.13 | 0.022 | 0.15 |
| E-I ratio | 1.19±0.12 | 1.19±0.11 | 0.59 | 0.14 | 0.11 |
| SD (bpm) | 4.5±2.3 | 4.4±2.0 | 0.31 | 0.56 | 0.25 |
| MCR | 1.95±1.21 | 1.92±1.24 | 0.30 | 0.18 | 0.42 |
| RMSSD (bpm) | 54.2±39 | 56.9±39 | 0.16 | 0.40 | 0.49 |
|  |  |  |  |  |  |
| Low E-I median (%) | 11.9 | 10.7 | 0.71 | 0.17 | 0.37 |
| Low E-I mean (%) | 11.9 | 10.2 | 0.51 | 0.96 | 0.61 |
| Low E-I ratio (%) | 11.1 | 8.2 | 0.09 | 0.84 | 0.79 |
| Low SD (%) | 12.7 | 10.9 | 0.44 | 0.85 | 0.84 |
| Low MCR (%) | 8.8 | 11.6 | 0.03 | 0.039 | 0.04 |
| Low RMSSD (%) | 12.7 | 8.2 | 0.006 | 0.11 | 0.99 |

_* P 1 adjusted for age and sex_

_** P 2 adjusted for age, sex, season, current smoking, diabetes, and BMI_

_***P 3 adjusted for age, sex, season, current smoking, diabetes, BMI, and heart rate_

_1. p-values were calculated with linear regression with continuous measure of HRV as dependent variable_

_2. p-values were calculated with logistic regression for low HRV (i.e. HRV in lowest 10% of distribution in population)_

_48 individuals were both in Q5 of % sedentary time and in Q5 of % moderate to vigorous activity and were therefore exclude_Supplementary table 6. Comparisons between different patterns of moderate-to-vigorous physical activity and sedentary time, divided into groups above and below median.

|  | Low moderate/vigorous activity | | High moderate/vigorous activity | | P 1^*^ | P 2^**^ | P3^***^ |
| --- | --- | --- | --- | --- | --- | --- | --- |
|  | High sedentary time | Low sedentary time | High sedentary time | Low sedentary time |  |  |  |
| Number 4125 | 1365 | 674 | 874 | 1412 |  |  |  |
| Age (years) | 57.7±4.3 | 57.9±4.2 | 57.0±4.2 | 57.3±4.3 |  |  |  |
| Women (%) | 53 | 72 | 41 | 58 |  |  |  |
| %sedentary time (accelerometer) | 62±6.3 | 45±5.6 | 59±4.6 | 43±6.6 |  |  |  |
| % moderate to vigorous time (accelerometer) | 3.4±1.3 | 3.9±1.1 | 7.9±2.0 | 9.2±3.2 |  |  |  |
| Systolic blood pressure (mmHg) | 125±17 | 123±16 | 123±16 | 122±16 |  |  |  |
| Diastolic blood pressure (mmHg) | 76.7±9.9 | 75.0±9.3 | 75.1±9.6 | 74.4±9.6 |  |  |  |
| Smoking (%) | 20 | 20 | 10 | 14 |  |  |  |
| Diabetes (%) | 10 | 7.4 | 7.9 | 5.7 |  |  |  |
| BMI (kg/m^2^) | 28.1±4.8 | 26.4±4.1 | 27.5±4.6 | 26.2±4.1 |  |  |  |
| LDL (mmol/l) | 3.6±1.0 | 3.6±0.94 | 3.6±0.97 | 3.6±0.92 |  |  |  |
| Heart rate (bpm) | 64.8±9.2 | 64.4±8.9 | 62.1±8.8 | 62.9±8.4 | <0.001 | <0.001 |  |
|  |  |  |  |  |  |  |  |
| Low E-I median (%) | 11.4 | 8.2 | 10.5 | 10.1 | 0.34 | 0.44 | 0.41 |
| Low E-I mean (%) | 11.1 | 7.6 | 10.6 | 9.6 | 0.23 | 0.27 | 0.23 |
| Low E-I ratio (%) | 10.6 | 6.1 | 7.7 | 7.8 | 0.004 | 0.094 | 0.14 |
| Low SD (%) | 11.9 | 8.8 | 11.3 | 9.9 | 0.34 | 0.58 | 0.49 |
| Low MCR (%) | 10.1 | 9.3 | 7.3 | 11.5 | 0.009 | 0.012 | 0.01 |
| Low RMSSD (%) | 11.9 | 7.9 | 8.8 | 8.4 | 0.010 | 0.19 | 0.22 |

^*P 1 p-value (3 degrees of freedom, df) adjusted for age and sex^

^**P 2 adjusted for age, sex, season, current smoking, diabetes, and BMI^

^***P 3 adjusted for age, sex, season, current smoking, diabetes, BMI, and heart rate^

Supplementary table 7. Odds ratios (ORs) and p-values with mutual adjustments for % sedentary time and % moderate to vigorous physical activity time from logistic regression models. Other covariates in the model were age, sex, season, current smoking, diabetes, and BMI. Dependent variables were low RSA or HRV from deep breathing test (i.e. lowest 10% of the population). A multiple linear regression model was used to analyze heart rate (dependent variable).

|  |  |  | | | |  |
| --- | --- | --- | --- | --- | --- | --- |
|  |  |  |  |  |  |  |
| **N=4,325** | **Sedentary time** |  |  | **Moderate to vigorous activity** |  |  |
|  | OR | 95% CI | P | OR | 95% CI | p |
| Low E-I median | 1.79 | 0.58-5.5 | 0.31 | 4.7 | 0.18-125 | 0.35 |
| Low E-I mean | 1.89 | 0.60-5.9 | 0.28 | 1.30 | 0.044-38 | 0.88 |
| Low E-I ratio | 1.73 | 0.50-6.0 | 0.39 | 0.20 | 0.005-8.6 | 0.40 |
| Low SD | 2.2 | 0.72-6.6 | 0.17 | 1.5 | 0.056-40 | 0.81 |
| Low MCR | 0.37 | 0.12-1.1 | 0.08 | 0.58 | 0.021-16 | 0.75 |
| Low RMSSD | 2.16 | 0.67-6.9 | 0.20 | 0.10 | 0.003-3.8 | 0.22 |
|  |  |  |  |  |  |  |
|  | Beta | Standard error of beta | p | Beta | Standard error of beta | p |
| Heart rate (per 10 bpm) | -0.001 | 0.001 | 0.61 | -0.026 | 0.005 | <0.001 |

_ORs are expressed as proportions of sedentary time and moderate to vigorous time out of total wear time._

_Heart rate was analysed as a continuous variable (per 10 bpm increment) using multiple linear regression._
